# Skull Vibration-Induced Nystagmus Test in a Human Model of Horizontal Canal Plugging

**DOI:** 10.3390/audiolres11030028

**Published:** 2021-06-24

**Authors:** Georges Dumas, Christol Fabre, Anne Charpiot, Lea Fath, Hella Chaney-Vuong, Philippe Perrin, Sébastien Schmerber

**Affiliations:** 1 Department of Oto-Rhino-Laryngology, Head and Neck Surgery, University Hospital, 38043 Grenoble, France; georges.dumas10@outlook.fr (G.D.); cfabre2@chu-grenoble.fr (C.F.); 2 EA 3450 DevAH, Development, Adaptation and Handicap, Faculty of Medicine, University of Lorraine, 54500 Vandoeuvre-lès-Nancy, France; philippe.perrin@univ-lorraine.fr; 3 Service d’ORL et CCF, Aveue Molière, Hôpital de Hautepierre, Hôpitaux Universitaires de Strasbourg, CEDEX, 67098 Strasbourg, France; Anne.Charpiot@chru-strasbourg.fr (A.C.); lea.fath@chru-strasbourg.fr (L.F.); hella.vuong@chru-strasbourg.fr (H.C.-V.); 4 BrainTech Lab INSERM UMR 2015, 38043 Grenoble, France; 5 Otologie, Neuro-Otologie, Implants Auditifs, Centre d’Implantation Cochléaire des Alpes, Clinique Universitaire Oto-Rhino-Laryngologie, Université Grenoble Alpes, INSERM UMR 1205, CHU A. Michallon BP 217, CEDEX 09, 38043 Grenoble, France

**Keywords:** skull vibration-induced nystagmus, horizontal canal plugging, disabling Menière’s disease, caloric test, video head impulse test

## Abstract

Background/Aim: the aim of this study was to assess the skull vibration-induced nystagmus test (SVINT) results and vestibular residual function after horizontal semicircular canal (HSCC) plugging. Methods: In this retrospective chart review performed in a tertiary referral center, 11 patients who underwent unilateral horizontal semicircular canal plugging (uHSCCP) for disabling Menière’s disease (MD) were included. The skull vibration-induced nystagmus (SVIN) slow-phase velocity (SPV) was compared with the results of the caloric test (CaT), video head impulse test (VHIT), and cervical vestibular-evoked myogenic potentials (cVEMP) performed on the same day. Results: Overall, 10 patients had a strong SVIN beating toward the intact side (Horizontal SVIN-SPV: 8.8°/s ± 5.6°/s), 10 had a significant or severe ipsilateral CaT hypofunction, 10 had an ipsilateral horizontal VHIT gain impairment, and 3 had altered cVEMP on the operated side. Five had sensorineural hearing worsening. SVIN-positive results were correlated with CaT and horizontal VHIT (HVHIT) results (*p* < 0.05) but not with cVEMP. SVIN-SPV was correlated with CaT hypofunction in % (*p* < 0.05). Comparison of pre- and postoperative CaT % hypofunction showed a significant worsening (*p* = 0.028). Conclusion: SVINT results in a human model of horizontal canal plugging are well correlated with vestibular tests exploring horizontal canal function, but not with cVEMP. SVINT always showed a strong lesional nystagmus beating away from the lesion side. SVIN acts as a good marker of HSCC function. This surgical technique showed invasiveness regarding horizontal canal vestibular function.

## 1. Introduction

**Figure 1:**
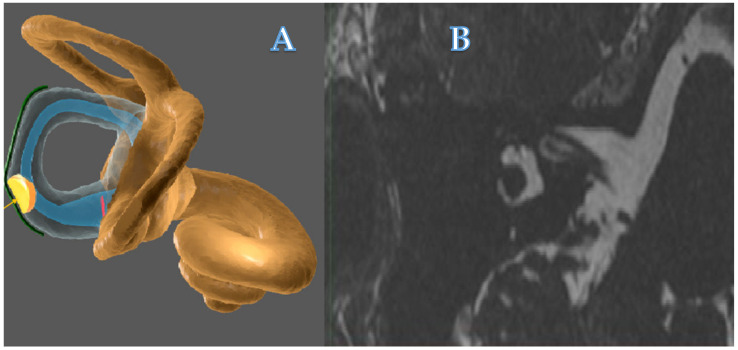
(**A**) Conceptual imaging of HSCC bone plugging. (**B**) MRI T2 picture of postsurgical state with interrupted HSCC. Right labyrinth.

Horizontal canal plugging has recently been described in disabling Menière’s disease (MD) treatment in selected patients with severe pure rotatory attacks of vertigo in the horizontal plane [1]. This supposed minimal invasive surgery aims at diminishing horizontal semicircular canal (HSCC) function and ablate endolymphatic liquid movement while preserving anatomical integrity of inner ear sensory cells (Figure 1). This technique intends to be etiopathogenic and not destructive. The caloric test (CaT) and video head impulse test (VHIT) need the integrity of inner ear hair cells and mobilization of cupula by endolymphatic liquid in the horizontal or another semicircular canal (SCC).

The skull vibration-induced nystagmus test (SVINT) in severe or total unilateral vestibular lesion patients usually reveals a strong (slow-phase velocity around 10°/s) vibration-induced nystagmus (VIN) beating away from the lesion side [2,3,4]. In animals, skull vibrations or bone-conducted vibrations stimulate type I inner ear hair cells of both canal and otolith structures at 100 Hz [5] and phase-locked AP responses are recorded on irregular discharging neural fibers. At such high frequencies type I inner ear hair cells and hair bundles are directly mobilized by the liquid wave induced by vibrations, as suggested by Curthoys [5,6,7]. In addition, SVINT stimulation uses frequencies higher than 60 Hz, which do not implicate the mobilization of SCC cupula [8]. The type I inner ear hair cells are located at the crest of the cupula and are more sensitive and vulnerable to forces exerted on the endolymphatic liquid than type II inner ear hair cells located at the base of cupula [5].

Some vestibular tests explore with more or less selectivity the horizontal canal function: the caloric test shows HSCC cupula’s capability to respond to canal liquid mobilized by convection at different temperatures for low-frequency stimulations and concerns type II inner ear hair cells (IEHC). VHIT shows canal cupula’s capability to respond to high-frequency mobilizations (type I IEHC). SVINT directly stimulates the hair bundle of type I inner ear hair cells of otolith and canal structures by bone conduction at 100 Hz in animals [5]. The first aim of this study was to assess the possible correlation between SVIN, HSCC vestibular tests, or otolith saccular exploration tests in a human model of horizontal canal exclusion. The second aim was to analyze the surgical technique invasiveness regarding vestibular function.

## 2. Materials and Methods

### 2.1. Population

In total, 11 patients, 5 women and 6 men, with the mean age of 57 years old (34–78), who were operated on with unilateral horizontal semicircular canal plugging (HSCCP) for intractable unilateral MD from 2006 to 2016 were included in this study. They corresponded to criteria of definite MD (Barany Society 2015) and grading among saved data were considered. They had no previous surgery for their MD. All patients had severe disabling vertigos for more than 6 months (more than 1 crisis/month with vomiting) before surgery. They were included for surgery with symptoms as previously described by Charpiot et al. [1]: fluctuating hearing loss associated with pure rotatory vertigos in the horizontal canal plane.

All participants gave informed consent before data collection, in accordance with the current ethical laws, and all experiments were performed in accordance with the Helsinki II Declaration.

### 2.2. Methods

All the vestibular tests and pure tone audiometry (PTA) were evaluated on the same day post operatively. CaT and audiological exploration tests were performed before and after surgery.

#### 2.2.1. Skull Vibration-Induced Nystagmus Test (SVINT)

The vibratory stimulation was applied in all participants successively to the vertex and the right and left mastoid processes (i.e., at the level of the external auditory canal) using the VVib 3F (Synapsys, Marseille, France) at 100Hz. The hand-held vibrator was applied for 10 s. The force of application evaluated in a previous work is about 10 N at 100 Hz [9].

Participants were in an upright sitting position. The participants’ head was held in place by one of the examiner’s hands. The tip of the vibrator was positioned perpendicular to the skin and held in position by the examiner’s other hand. A 2D VNG recording (i.e., eyes open behind a videoscopic helmet, Synapsys, Inc., France) was used to record the possible VIN occurrence and its slow-phase eye velocity (SPV, in °/s).

The test was considered positive on mastoid locations when the VIN was reproducible, started with the stimulation, beat toward the same side (after right or left mastoid stimulation) with SPV higher than 2.5°/s, and disappeared upon stimulation withdrawal without secondary nystagmus reversal [4]. The same characteristics were applied to vertex stimulations. When a discordant VIN direction between vertex or mastoid stimulations was observed, only the VIN direction with concordant result in two locations was considered.

Results were given in a quantitative evaluation (slow-phase velocity (SPV) was expressed in °/s and in a semi quantitative formulation: + corresponded to a low response correlated with a SPV value lower than 5°/s but higher than 2.5°/s; ++ corresponded to a strong response with a SPV lower than 10°/s but higher than 5°/s; +++ corresponded to a very strong response with a SPV > 10°/s. All patients were quoted under videoscopy with crosses and their components (horizontal, vertical) were analyzed.

#### 2.2.2. Caloric Test (CaT)

The test was performed using VNG according to the bithermal caloric test protocol of Fitzgerald-Hallpike [10] with water infusion of the ear canal for 30 s. A hypofunction >20% (using Jongkees formula) was considered significant. Values between 20% and 80% corresponded to mild hypofunction and values >80% were considered as severe hypofunction. Areflexia was defined when CaT showed no responses even at 15 °C cold water.

#### 2.2.3. Head Shaking Test (HST)

The examiner shook the patient’s head in the horizontal canal plane (head flexed to 30°) for 20 s at a frequency of about 2 Hz, and rotation amplitude of 45° on both sides and responses were analyzed under VNS. The test was considered positive when more than one head shaking nystagmus was observed [11].

#### 2.2.4. Video Head Impulse Test (VHIT)

This test was performed on a sitting upright subject with the VHIT device Ulmer- Synapsys (Marseille, France) at 100 Hz image sampling. This device records eye position and shows possible covert and overt saccades. It also analyzes the gain of the vestibulo-ocular reflex (VOR). The examiner exerted head impulses (10–20° of amplitude) with a velocity higher than 200°/s in the horizontal, anterior, and posterior SCC planes. The test was considered positive for a canal when the VOR gain was <0.8 for the HSCC or a covert or overt saccade was observed. A gain <0.68 was considered positive for the posterior and anterior canals [12]. Severe hypofunction was defined when the gain value was <50%. A VHIT gain asymmetry >20% was considered significant.

#### 2.2.5. Cervical Vestibular-Evoked Myogenic Potentials (cVEMP)

Patients were positioned supine on a stretcher and were asked to turn their head contralaterally to the stimulated ear to obtain a maximal activation of the ipsilateral sternocleidomastoid muscle. Self-adhesive electrodes were used to record surface electromyographic from symmetrical locations at the junction of the superior one-third and inferior two-thirds of both sternocleidomastoid muscles. The reference electrode was located over the upper sternum and the ground electrode was positioned on the medium part of the forehead. The latency of the two early waves was measured in ms. Peak-to-peak amplitude between P13 and N23 was measured in mV. Stimulations were delivered with an earphone (calibrated TDH 39 headphones) in each ear and used clicks at 100 µs, and 200 stimulations were averaged; cVEMPs were recorded with the Racia Centor C device (Racia Biomedical Inc., Bordeaux, France), the filter band was 16−1600 Hz. The minimal threshold to obtain an identifiable response was noted. The test was considered positive and showing a hypofunction when the cVEMP response was abolished or thresholds were augmented (25 dB higher than the intact side) or when the difference in amplitude peak to peak (P13-N23) between both sides was higher than 50%.

#### 2.2.6. Audiogram and Subjective Scales

PTA was performed with the Madsen Astera device (Otometrics, Taastrup, Denmark) and the mean of 500, 1000, 2000, and 4000 Hz frequencies determined the degree of hearing loss (Internation Bureau for Audiophonology). Patients were given a vertigo subjective scale to fill out (DHI and AAO HNS vertigo scale).

### 2.3. Statistical Analysis

The McNemar test was used to compare results of the different tests (SVINT, CaT, HST, HVHIT, cVEMP) in the population. The Kendall rank correlation coefficient and linear regression were used for quantitative score of VHIT and CaT. A *p*-value <0.05 was determined as significant.

## 3. Results

### 3.1. Vestibular Results

**Figure 2:**
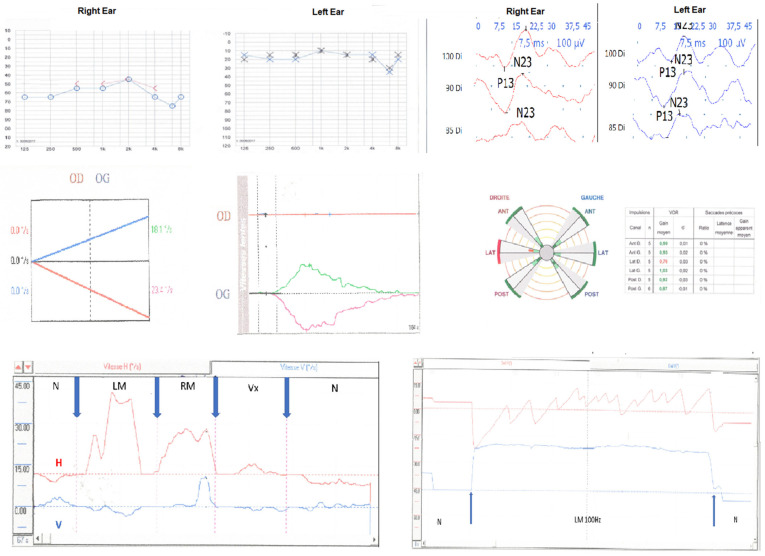
Example of vestibular test results for a typical patient operated on the right side. From up to down and left to right: PTA: moderate hearing loss on the right ear; preservation of cVEMP responses; caloric test: right caloric areflexia; VHIT: isolated and moderate hypofunction of the sole horizontal semicircular canal (gain = 0.76); SVINT: strong SVIN beating toward the intact side (SVIN-SPV = 16°/s); H = horizontal component, V = vertical component (the vestibular AAO subjective scale score = 1; DHI score = 12).

Figure 2 shows the example of typical recordings. Table 1 shows vestibular results. 

SVINT was positive with a VIN beating toward the intact side in 10/11 cases (91%), with mean SPV value: 8.8°/s ± 5.6°/s. Very strong responses (VIN SPV > 10°/s) were observed in four cases, strong responses (VIN –SPV between 5 to 10°/s) in four cases, and low responses in two cases.

Only one case was negative and was associated with AAO HNS grade 4 and a DHI score at 50. In this case, the postsurgical MRI control confirmed the persistent plugging, the PTA on the operated ear had a normal threshold (the PTA threshold on the operated ear was not modified post operatively), and the cVEMPs were normal. VHIT showed a significant right canal hypofunction (gain = 0.50) and the CaT, which showed in the preoperative condition a right hypofunction at 54%, revealed a postoperative right hypofunction not significantly modified at 49%.

HST was positive with a head shaking nystagmus beating toward the intact side in 5/11 cases (50%), beating toward the lesion side in 1/12 cases, and negative in 5/11 cases.

VHIT was significantly impaired (gain lower than 80%) for the ipsilateral horizontal canal in 10/11 cases (91%). The case with a normal result corresponded with AAO HNS grade 3. Two cases had moderate HVHIT reduction (gain between 70% and 80%) and corresponded to AAO HNS scale grade 4. The horizontal canal hypofunction was severe with a gain <50% in seven cases (63% of cases).

The other canals were altered ipsilaterally in five cases for the posterior SCC (45% of cases) and in two cases for the anterior SCC (18%). The contralateral posterior SCC function was altered in five cases (hypofunction related to aging). No contralateral anterior SCC function was significantly modified.

Caloric test: One patient refused the CaT for the postoperative control. A postoperative ipsilateral significant hypofunction (>20%) was observed in 10/10 cases (100% of cases. Height patients (60%) had a severe hypofunction >80% (among which six had an areflexia at very cold water of 15 °C).

The rotatory test showed in 8/11 patients a normal result without significant vestibular preponderance. Additionally, 3/11 cases (27%) had a vestibular preponderance either toward the operated side (2 cases) or toward the intact side (1 case). No clear correlation was observed with the result on quality of life (QOL) tests.

Postoperative cVEMP were altered in 4/11 cases (36%) with an ipsilateral hypofunction in 3 cases (27%) and a contralateral hypofunction in 1 case (9%).

### 3.2. Correlations between the Different Vestibular Tests

Results of the McNemar test with continuity correction revealed:

- VIN beating contralaterally vs. CAT ipsilateral hypofunction: *p* > 0.99 (the tests are identical);
- VIN beating contralaterally vs. VHIT ipsilateral hypofunction: *p* > 0.99 (the tests are identical);
- VIN beating contralaterally vs. cVEMP ipsilateral hypofunction: *p* = 0.02 (the two tests are different);
- VIN beating contralaterally vs. HST positivity: *p* = 0.62 (the two tests are identical).

**Figure 3:**
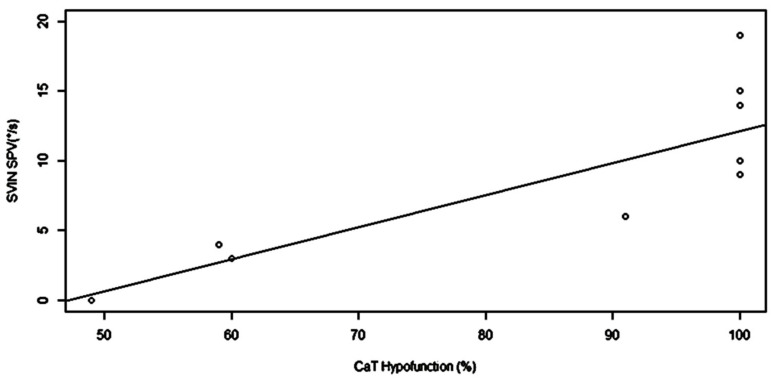
Regression graph between SVIN-SPV (horizontal canal component) and caloric test (CaT). Hypofunction in %. Statistical results are as follows: beta = 0.23067; standard error = 0.05647; *p* = 0.00351; R^2^ = 0.63. The SVIN-Hor SCC is correlated with the caloric test, which is a marker of the HSCC function.

The Kendall coefficient between SVIN-SPV and CaT hypofunction in % was 0.77 and the linear regression is shown in Figure 3 (β = 0.23, SE = 0.06, *p* = 0.003).

No significant correlation was observed between SVIN-SPV and HVHIT % of asymmetry: τ = 0.09, linear regression: *p* = 0.9).

### 3.3. Pre- and Postoperative Audiometry and Caloric Tests

**Figure 4:**
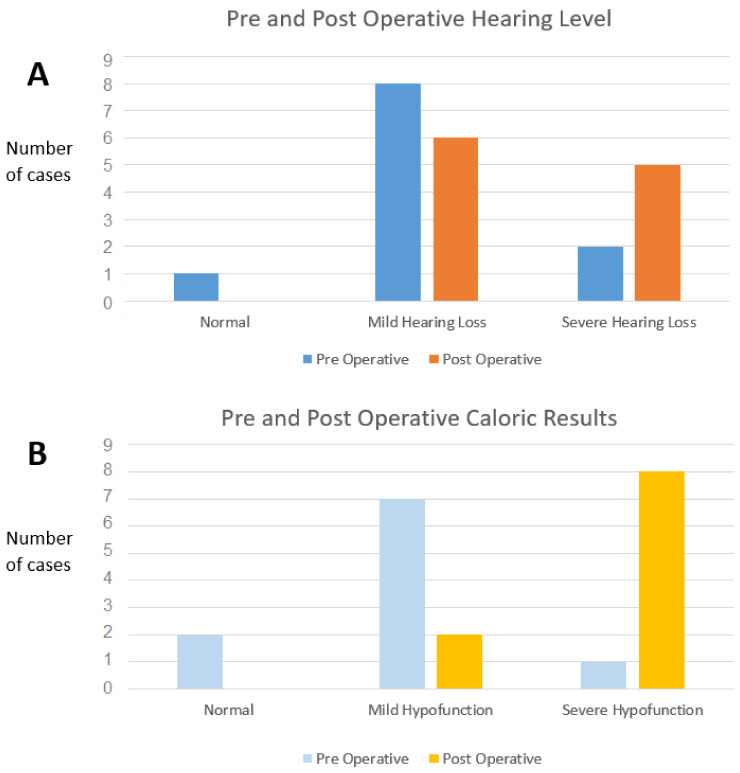
Pre- and post-surgery results with caloric test and pure tone audiometry (PTA). (**A**): PTA (n = 11): three cases are impaired with severe post-surgery hearing loss (18%). (**B**): Caloric test (n = 10): no areflexia was observed before surgery. After surgery, a severe hypofunction (CaT hypofunction >80%) was observed in 82% of cases.

PTA thresholds worsening of more than 15 dB was observed in 5/11 cases (45%). A major PTA thresholds worsening was observed in three cases (i.e., transformation from normal hearing or mild hearing loss to severe hearing loss). One patient had a postoperative total deafness (Figure 4A), Wilcoxon paired sign rank test did not assess statistically significant changes with a *p*-value = 0.06.

The CaT was performed pre- and post-surgery in all the patients except one, who refused the examination for the control test (Figure 4B).

The CaT showed significant modification; severe hypofunction (Jongkees formula >80%) was never observed in preoperative patients but was measured in eight operated on patients (67% of cases) among whom six had a caloric areflexia (Figure 4B). The comparison between pre- and postsurgical CaT hypofunction in % showed a significant worsening (*p* = 0.028).

### 3.4. Subjective Scales

Postoperative AAO subjective scale score median was 2 [1;2.75] and it was 12 [5;42.5] for the DHI score.

## 4. Discussion

The altered results of HVHIT here observed contrast with the usual normality [13] or seldom modified results [14] for VHIT described in non-operated MD patients. The technique of the lateral canal plugging was aimed initially at preserving inner ear hair cell functionality among endolymphatic structures and was therefore supposed to be a mini-invasive surgical procedure. However, our results indicate often severe alteration of HSCC function as well for CaT, SVIN, or HVHIT, suggesting that the procedure is in fact more invasive than expected.

This severe (in 60% of cases) or moderate (in 38%) impairment of the HSCC function measured at the VHIT after surgery is confirmed by pre- and postoperative CaT results observed in our series. CaT preoperative results showed normal function to moderate hypofunction in 90% of cases and no areflexia. Conversely, 100% of operated patients showed a significant caloric hypofunction, 80% had a severe hypofunction higher than 80%, and six cases had an areflexia. In the literature, CaT is modified in non-operated Menière disease patients in between 53% and 76% of cases with a moderate hypofunction [14,15,16,17].

This strong ipsilateral postoperative CaT hypofunction observed in all cases may result either from a functional modification related to the absence or strong diminution of thermic endolymphatic liquid convection following the Barany conception [12], direct alteration of type II hair cells, or both mechanisms. McGarvie et al. suggested that the caloric hypofunction observed in non-operated MD may result from an hydropic expansion of the lateral canal membranous labyrinth, which permits convective recirculation within the duct that allows for the dissipation of the hydrostatic force that would normally cause cupular displacement and nystagmus in the caloric test [18].

Paige G. [19] described caloric vestibular responses despite canal inactivation by HSCC plugging. This suggests strongly that the post-surgery CaT profound impairment may partly be the result of the diminution of endolymphatic liquid mobilization, but more probably directly related to postsurgical alteration of inner ear hair cells.

In non-operated MD, the moderate CaT hypofunction usually described in the literature is associated most often with preservation of a normal VHIT function.

The HVHIT results’ alterations after HSCC plugging may be related to the absence of endolymph mobilization [20] induced by canal blockage and may reveal a pure functional failure of the canal without necessary hair cell impairment. However, semicircular canal plugging is highly effective in blocking sensitivity of individual canals [21], at least at moderate angular motion stimuli, but this effectiveness does not extend to stimuli involving high accelerations (>2 Hz). A residual response persists even after complete occlusion of the duct at high-frequency stimulations [21]. Thus, HVHIT should not be so profoundly impaired as it was in our results in the hypothesis of a non-invasive technique. Hence, we suspect that this impairment is due to an associated histological lesion of HSCC inner ear hair cells, albeit we did not observe a tight correlation between horizontal SVIN-SPV and HVHIT gain asymmetry as observed by Battuecas et al. in vestibular neuritis [22]. However, VHIT alterations appear less severe than CaT (HVHIT ipsilateral gain with moderate alteration in 38% of cases) and the VHIT % of asymmetry was normal or moderate in 60% of cases.

The severe alteration of SVIN in our postoperative patients contrasts with the results usually observed in non-operated MD patients in the literature. Authors have signaled a not frequent occurrence of a positive SVINT (28% to 64%) depending on the proximity of a vertigo spell [20,23].

In larger series of non-operated MD, it was demonstrated that SVIN may be observed in 59% to 66% of cases [24,25] and usually shows a low SPV < 4°/s (3.89 ± 3.9°/s) [25].

SVIN usually reflects the direct alteration of inner ear hair cells. We hypothesize that the hyper pressure induced by the plugging of the HSCC may alter type I hair cells located at the crest of cupula. Type II inner ear hair cells located at the ampulla base are supposed to be less solicited and mobilized by endolymph pressure variations than type I inner ear hair cells during the surgical procedure [5,12]. The liquid mobilization in a blocked HSCC is limited and less important than on the intact side, showing a vestibular asymmetry related to a less important cupula mobilization. However, at such high frequencies of 100 Hz, SVIN stimulation of horizontal SCC hair cells is not related to the classical cupula mechanical mobilization, which has a cut off frequency of 63 Hz [8], but it is related to the direct stimulation of type I inner ear hair cells bundle as suggested by Curthoys [7]. A relative preservation of SVIN responses could have been expected, but in contrast, SVIN strong positivity observed in our patients suggests more a direct traumatic alteration (lesion) of hair cells than a pure functional defect.

In our series only one case, associated with actual persisting plugging verified by MRI, demonstrated this technique as mini-invasive with the absence of SVIN, normal hearing, no postoperative CaT hypofunction modification, and normal cVEMP, suggesting the integrity of cupula inner ear hair cells.

Our results confirm that SVINT is a good indicator of lateral canal function. The SVINT results are well correlated with HVHIT and the CaT, the gold standard test of the function of the horizontal SCC. This test is as sensitive as CaT to signal horizontal canal dysfunction in this series of patients explored postoperatively.

SVIN acts as a vestibular Weber test and shows a vestibular asymmetry [2,3,4] at least for the horizontal SCC function.

In this model, SVIN results were not correlated to cVEMP, which respond mainly to Saccule stimulations. The prevalence of cVEMP alteration in non-operated MD is around 30% in the literature [26], which is similar to the cVEMP results in our operated patients, suggesting that this surgical technique does not modify otolith function.

SVIN has been proposed as a global vestibular test that involves both canal and otolithic structures at 100 Hz [4]. In animals, bone-conducted vibrations (BCV) are signaled to stimulate directly the hair bundle of both canal and otolith type I inner ear hair cells and induce phase-locked responses from irregular discharging afferent neurons with less energy needed for otolith system stimulation than for canal structures (0.2 g vs. 2 g, respectively [5,7]. However, the induced nystagmus is more relevant from a primarily canal contribution at 100 Hz. The predominant canal contribution over otolith structures to provoke a SVIN at this frequency has been documented in animals by Dlugaiczyck et al. [27] and already suggested in human by Dumas et al. [28].

Discordant results are usually observed between CaT and HVHIT in non-operated MD, as suggested by McGarvie et al., who considered the integrity of hair cells responding normally to cupular displacement or sollication by head mobilization, but that they are less stimulated by the CaT temperature difference since the thermic diffusion stimulation is less efficient in case of hydropic labyrinth [12]. However, this discrepancy disappeared in our human model of canal exclusion, which shows a frequent modification of HVHIT gain but not of cVEMP, suggesting invasiveness of the surgical technique for the horizontal SCC function.

Our study presents limitations: our population was limited to 11 patients, but such a surgery is seldom performed and constitutes a unique model of human HSCC exclusion. In this presentation, CaT was performed before and after surgery, but SVIN was only performed after surgery.

The correlation between SVIN with VHIT was only studied for the horizontal component and not for the vertical and torsional components.

## 5. Conclusions

The skull vibration-induced nystagmus test, a global vestibular test that stimulates both canal and otolith structures, was demonstrated to be well correlated in clinical analysis with the horizontal canal vestibular tests in a human model of horizontal semicircular canal plugging. It is as sensitive as the caloric test and horizontal video head impulse test. It is a good indicator of horizontal semicircular canal unilateral dysfunction. The HSCC plugging technique is not a minimally invasive procedure.

## Figures and Tables

**Table 1:** Population. Audiological and Vestibular Results. Subjective Scales Results.

Patient	Gender	Age	Lesion Side	Hearing Loss		Subjective Scales Post Op		Caloric Test Ipsi lat Hypofunction (%)		L-VHIT Post Op(%)		HST Direction Postop	cVEMP Hypofunction Postop	SVIN-SPV Postop (°/s)
				Preop.	Postop.	AAO	DHI	Preop.	Postop.	Gain Ipsilat	Asymmetry			
1	M	78	L	Mild	Sev	1	6	NA	NA	21	72	R	L	8
2	F	52	R	Mild	Sev	1	12	77	100	76	26	L	N	9
3	F	59	L	Mild	Mild	4	46	92	100	62	38	L	N	15
4	M	50	R	N	Mild	1	18	62	91	73	25	L	R	6
5	M	45	R	Mild	Sev	1	0	66	100	91	11	N	R	14
6	F	77	R	Mild	Mild	1	4	0	100	33	65	L	N	19
7	M	57	L	Mild	Mild	2	6	84	59	39	64	R	N	4
8	M	45	L	Sev	Anacusis	3	32	0	100	27	72	N	N	10
9	F	44	R	Mild	Mild	4	50	64	49	50	37	L	N	0
10	M	66	L	Mild	Sev	2	46	9	60	26	41	N	N	3
11	M	62	R	Mild	Sev	2	4	51	100	6	92	L	R	9
Test positivity (%)								70	100	91		73	27	91

## Data Availability

Not applicable.
